# Synthesis and Antioxidant Evaluation of Enantiomerically Pure Bis-(1,2,3-triazolylmethyl)amino Esters from Modified *α*-Amino Acids

**DOI:** 10.1155/2014/264762

**Published:** 2014-10-15

**Authors:** Juan I. Sarmiento-Sánchez, Adrián Ochoa-Terán, Ignacio A. Rivero

**Affiliations:** ^1^Facultad de Ingeniería, Universidad Autónoma de Sinaloa, Boulevard de las Américas S/N, 80040 Culiacán, SIN, Mexico; ^2^Centro de Graduados e Investigación del Instituto Tecnológico de Tijuana, Boulevard Alberto Limón Padilla S/N, Apdo. Postal 1166, 22500 Tijuana, BC, Mexico

## Abstract

The efforts for synthesis of enantiomerically pure bis-(1,2,3-triazolylmethyl)amino esters **6** are reported in good yields from an *in situ* generated *α*-azidomethyl ketone. Optimum experimental conditions were established for preparation of *α*-halomethyl ketones **10** and *α*-*N,N*-dipropargylamino esters **11**, all derived from *α*-amino acids. The starting materials reacted under conventional click chemistry conditions, revealing a specific reactivity of bromomethyl ketones over chloromethyl ketones. The antioxidant activity of compounds **6** was assayed by DPPH method. The compound **6c** with an IC_50_ of 75.57 ± 1.74 *μ*g mL^−1^ was the most active. Technically, this methodology allows the preparation of a combinatorial library of analogues with different structural characteristics depending on the nature of the modified *α*-amino acids employed in the synthesis.

## 1. Introduction

**Scheme 1:**
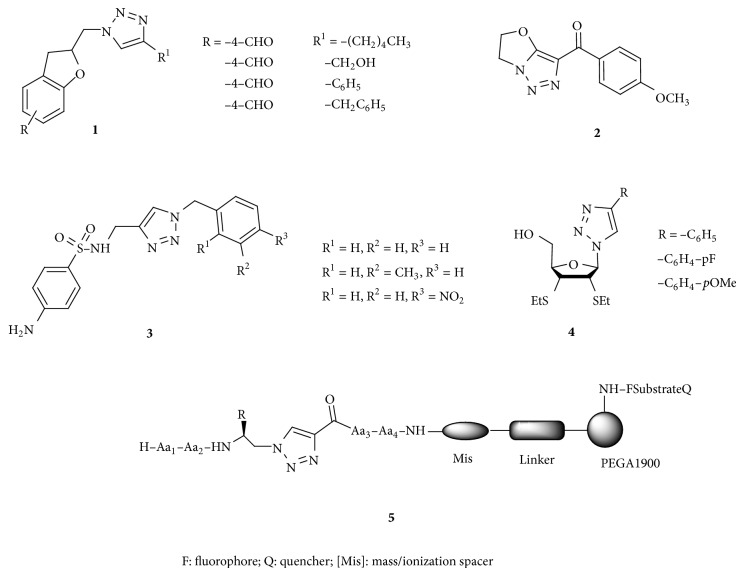


Click chemistry has been defined as an efficient and an almost perfect method (very selective, with high yields and wide scope) for the synthesis of new and diverse compounds based in a carbon-heteroatom bond formation. This reaction has been particularly useful for coupling two molecules, azides and alkynes, to get 1,2,3-triazole compounds [1]. However, it was only after the discovery of copper catalysis that its applications began to be studied [2]. Click chemistry meets the requirements of an innovative functionalization strategy for biomolecules because it is efficient, selective, and without side reactions. Rostovtsev et al. [3] and Tornøe et al. [4] have reported that 1,4-disubstituted 1,2,3-triazoles are specifically prepared from azides and terminal alkynes under copper(I) catalysis to give 1,4-substituted products with high regioselectivity. The regioisomeric 1,5-disubstituted triazoles are available from azides and terminal alkynes by the use of either magnesium acetylides or ruthenium catalysts [1, 5, 6]. 1,2,3-Triazole compounds have attracted attention because they exhibit a broad variety of biological activities. For example, compounds such as** 1** are active against* Mycobacterium tuberculosis* [7]; other compounds act as anticancer** 2** [8], antifungal** 3** [9], or antitumor agents** 4** [10], Scheme 1. Some biomedical applications have been described, for example, the labelling of biomolecules [11]. In addition, 1,2,3-triazoles have been used in the coupling of modified *α*-amino acids in oligopeptide synthesis [4, 12–17]. Furthermore, this class of modified oligopeptides** 5** showed activity as inhibitors of cysteine protease CPB2.8ΔCTE in* Leishmania mexicana* [18] and antiviral activity against HIV-1 gp120 [19, 20].

The cycloaddition of azides and alkynes is typically carried out in refluxing toluene, but labile molecules may not be stable under these conditions. Also, although organic azides are generally safe compounds, those of low molecular weight can be unstable and, therefore, difficult to handle. This is especially true for small molecules with several azide functionalities, which would be of much interest for the generation of polymeric structures. Thus, a methodology that avoids isolation of organic azides as intermediates is desirable. In literature have been reported some procedures to synthesize 1,2,3-triazoles where the organic azide is generated* in situ* [21–24].

Moreover, enantiomerically pure *α*-halomethyl ketones can be prepared from *α*-amino acids modifying the carboxylic group into a halomethyl group. Its selective reduction with sodium borohydride affords the* erythro*-amino alcohols as main products. As a result, these *α*-halomethyl ketones are often used as key intermediates in the stereoselective synthesis of biological active compounds [25]. In this sense, the N-acylated *α*-chloromethyl ketones are used as enzyme inhibitors [26, 27] and serve as precursors for the synthesis of the hydroxyethylamine isosteres, which are present in many of the inhibitors of angiotensin converting enzyme [28], renin [29], and HIV protease [30]. The *α*-chloromethyl ketones are typically converted to chlorohydrins and epoxides, which react with nucleophiles in the preparation of various enzyme inhibitors [25]. A typical reaction to prepare *α*-halomethyl ketones involves the conversion of N-acylated *α*-amino acid into *α*-diazoketone and a subsequent treatment with HX [31]. This procedure requires large amounts of diazomethane to produce *α*-halomethyl ketones; for this reason it is considered as an unsafe procedure. In recent years, the use of *α*-halomethyl ketones as intermediates in the synthesis of enantiomerically pure heterocyclic compounds, such as epoxides, azetidines, and aziridines, has been reported [32–35]. Furthermore, enantiomerically pure *α*-bromomethyl ketones can be prepared from *α*-amino esters and bromomethyllithium generated* in situ* [36, 37]. This procedure prevents the use of diazomethane and minimizes racemization.

On the other hand, there is an increasing interest in compounds with high antioxidants activities, which could counteract the oxidative stress associated with diseases. Antioxidant activity screening is commonly done by* in vitro* assays, such as the ABTS^•+^ [38], ferric-reducing [39, 40], and DPPH [41, 42] methods.

Recently, we have reported the synthesis of new enantiomerically pure compounds from chemical modifications of *α*-amino acids [43–45]. Continuing our research interest in triazoles chemistry [46, 47], now we report our efforts in the synthesis of new enantiopure 1,4-disubstituted 1,2,3-triazoles via conventional Huisgen cycloaddition using *α*-halomethyl ketones** 10** and *α*-*N,N*-dipropargylamino esters** 11** as one strategy for coupling in solution two enantiopure moieties derived from *α*-amino acids. From a combinatorial chemistry approach, the methodology developed in the present work could allow preparing a library of analogues, depending on the number and nature of *α*-amino acids synthetically modified. It is then feasible to expect a different behavior for each one of the possible products. We envisioned potential for these new bis-(1,2,3-triazolylmethyl)amino esters** 6** (Scheme 3) as biologically active substances and as building blocks for the syntheses of interesting modified oligopeptides.

## 2. Results and Discussion

### 2.1. Synthesis of Modified *α*-Amino Acids as Starting Materials for Click Chemistry

**Scheme 2:**
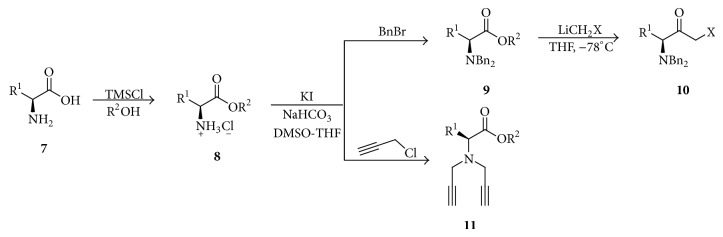
Synthesis of starting compounds from *α*-amino acids.

The synthesis of enantiopure *α*-halomethyl ketones** 10** and *α*-*N,N*-dipropargylamino esters** 11** can be achieved following the methodology depicted in Scheme 2. *α*-Amino acids** 7** were esterified with excellent yields using trimethylsilyl chloride (TMSCl) and methanol or ethanol as solvent. Then, compounds** 8** are alkylated under basic conditions using benzyl bromide to obtain* N,N*-dibenzylamino esters** 9**, which react with methyllithium chloride or methyllithium bromide* in situ* generated in order to obtain the enantiopure compounds** 10**. The *α*-halomethyl ketones synthesized only were characterized for NMR and immediately used for the next step in the reaction. Yields for the *α*-halomethyl ketones synthesized with this procedure are shown in Table 1.

For the synthesis of enantiopure dialkynes several *α*-amino acids were modified. The subsequent reaction of *α*-amino esters** 8** with propargyl chloride was carried out in order to obtain* N,N*-dipropargylamino esters** 11** with isolated yields between 59 and 87% (Table 2). The propargylamino esters** 11** and halomethyl ketones** 10** compounds were characterized for NMR and immediately were used for the synthesis of bis-(1,2,3-triazolylmethyl)amino esters.

### 2.2. Synthesis of Bis-(1,2,3-triazolylmethyl)amino Esters and Antioxidant Evaluation

**Scheme 3:**
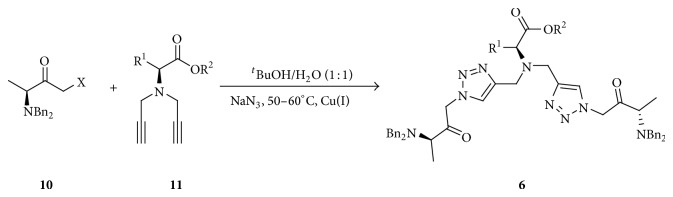
Synthesis of bis-(1,2,3-triazolyl) compounds using modified *α*-amino acids.

As is depicted in Scheme 3, the key process in the synthesis of bis-(1,2,3-triazole) compounds** 6** is the* in situ* generation of *α*-azidomethyl ketones [48, 49] by the reaction of sodium azide with *α*-halomethyl ketones** 10**. First, the synthesis of bis-(1,2,3-triazole)** 6a** was studied as model reaction in order to establish the optimum time required to complete the reaction. The bromomethyl ketone** 10e** and dialkyne** 11a** were used as starting materials in aqueous* tert*-butyl alcohol (^*t*^BuOH/H_2_O 1 : 1 v/v). The reaction was monitored during seven days by TLC. After this time starting materials were still present in the mixture. After reaction workup the product was isolated with 25% yield. Thus, based on our experience in the synthesis of 1,2,3-triazoles [46, 47], the temperature reaction was increased to 60°C and the product was identified by TLC after 12 h and at 48 h the product** 6a** was isolated with 65% yield.

Subsequently, four bis-(1,2,3-triazolylmethyl)amino esters** 6** were synthesized by thermally inducing the Huisgen cycloaddition reaction between ketone** 10e** and dialkynes** 11**. A temperature of 60°C and a time of 48 h were the optimal conditions for the synthesis of the products. The isolated product yields were in the range from 55 to 76%, depending on the dialkyne used as starting material (Table 3). The higher yield obtained for product** 6b** could be explained considering the role of the indole ring when the substrate interacts with copper(I) ion stabilizing the complex for longer time and favoring the formation to diacetylides in click reaction (Scheme 3). When bromomethyl ketone** 10f** was used for the reaction, the products were detected by TLC and ESI-MS, but it was not possible to isolate them by chromatography using alumina or silica gel.

Chloromethyl ketones** 10a–d** were also studied, but the expected products were not detected by TLC or isolated after workup. We assumed that, as chloromethyl ketones are less reactive than bromomethyl ketones, the formation of azides was not achieved and in consequence neither was the cycloaddition process.

It is noteworthy that in the ^1^H-NMR spectra of compounds** 6a–d** some signals appeared as overlapping peaks due to the similarity of the chemical environment of the hydrogens in the molecule, however, is possible to distinguish two signals between 5 and 6 ppm corresponding to the hydrogens of methylene attached to the triazole and the ketone carbonyl groups. The signal for triazole hydrogen appears at 7.22 ppm overlapping with other aromatic hydrogens. In the ^13^C NMR spectra the significant signals appear at 203 ppm for the ketone carbonyl, at 174 ppm for ester carbonyl, and at 146 ppm and 124 ppm for the two carbons of triazole ring. In the ESI-MS analysis the quasimolecular ions for all products were detected. These spectroscopic data are clear evidence supporting the formation of the assigned products.

In the antioxidant activity, gallic acid, ascorbic acid, and BHT are commonly used as reference standards in a comparative evaluation of antioxidant properties of new molecules [50]. Thus, the bis-(1,2,3-triazoles)amino esters** 6** were assayed up to 100 *μ*g mL^−1^ and presented less activity than reference standards (Table 3). The compound** 6c** showed the higher activity at 75.57 ± 1.74 *μ*g mL^−1^ being 4.2-fold and 10.2-fold more active than** 6a** and** 6b**, respectively.

## 3. Conclusions

In this work we have developed a methodology for the synthesis of enantiomerically pure bis-(1,2,3-triazolylmethyl)amino esters in good yields. Also, the* in situ* formation of organic azides from* N,N*-dibenzylamino *α*-halomethyl ketones as a key intermediate was implemented. An easy methodology was used to synthesize new dialkynes derived from *α*-amino acids. The results gave significant information about the specificity of the reactivity of halomethyl ketones for the synthesis of these new materials, those containing bromine being the most suitable. The developed methodology allows preparation of a combinatorial library of analogues with different structural characteristics depending on the nature of the modified *α*-amino acids employed in the synthesis.

## 4. Experimental Section

### 4.1. General Procedures

All reagents were purchased in the highest quality available and were used without further purification. The solvents used in column chromatography were obtained from commercial suppliers and used without distillation. Infrared spectra (FTIR) were recorded on a Perkin Elmer FT-IR 1600 spectrophotometer. Nuclear magnetic resonance ^1^H (at 200 MHz) and ^13^C (at 50 MHz) spectra were recorded on a Varian Mercury 200 MHz Spectrometer in CDCl_3_ with TMS as internal standard. ESI-MS spectra were obtained with an ion trap, and the intensities are reported as a percentage relative to the base peak after the corresponding *m*/*z* value. HRMS was recorded on an ESI/APCI-TOF Bruker model MicroTOF-II-Focus at the Universidad Autónoma Metropolitana, Campus Iztapalapa. Melting points were obtained on an Electrothermal 88629 apparatus.

### 4.2. General Procedure for the Synthesis of Bis-(1,4-disubstituted-1,2,3-triazoles)

To a solution of *α*-halomethyl ketone (2 equiv.) in* tert*-BuOH/H_2_O (4 mL 1 : 1 v/v) were added sodium azide (2 equiv.), dialkyne (200 mg, 1 equiv.), copper(II) sulfate pentahydrate (5% mol), and sodium ascorbate (10% mol), with vigorous stirring at 60°C for 48 h. The reaction mixture was then filtered through diatomaceous earth and silica gel under reduced pressure and then extracted with ethyl acetate (4 × 20 mL). The extracts were combined and dried over anhydrous sodium sulfate. After evaporation of the solvent, the resulting oil was purified by flash chromatography.

Methyl (*S*)-2-[bis-[[1-[(S)-3-(dibenzylamino)-2-oxobutyl]-1*H*-1,2,3-triazol-4-yl]methyl]-amino]propanoate (**6a**). Yield 65%; pale yellow solid; [*α*]^20^_*D*_ = −14.3 (*c* = 1.33 in MeOH); ^1^H NMR (CDCl_3_, 200 MHz): *δ* 7.47–7.04 (m, 22H), 5.66 (d, 2H, *J* = 18.4 Hz), 5.09 (d, 2H, *J* = 18.4 Hz), 3.99–3.39 (m, 18H), 1.43–1.06 (m, 9H); ^13^C NMR (CDCl_3_, 75 MHz): *δ* 202.9, 174.3, 146.0, 138.2, 128.9, 128.8, 127.7, 124.6, 61.4, 56.4, 55.6, 54.9, 51.5, 45.6, 15.5, 6.0. IR (KBr, pellet): 3144, 1734, 1654, 1602, 1454, 1148 cm^−1^. ESI-MS *m*/*z*: 796 [M+H]^+^, 818 [M+Na]^+^. HRMS calculated for C_46_H_54_N_9_O_4_ (MH^+^) 796.4293; found: 796.4294.

Methyl (*S*)-2-[bis[[1-[(S)-3-(dibenzylamino)-2-oxobutyl]-1*H*-1,2,3-triazol-4-yl]methyl]-amino]-3-(1*H*-indol-3-yl)propanoate (**6b**). Yield 76%; grey solid; m.p. 205–207°C; [*α*]^20^_*D*_ = −37.0 (*c* = 0.88 in CH_2_Cl_2_); ^1^H NMR (CDCl_3_, 200 MHz): *δ* 8.24–6.52 (m, 27H), 5.74–5.58 (dd, 2H, *J* = 16.2, 14.7 Hz), 5.48–5.33 (dd, 2H, *J* = 12.0, 11.9 Hz), 5.09–4.92 (dd, 2H, *J* = 14.5, 18.5 Hz), 3.99–3.20 (m, 20H), 1.20 (s, 6H); ^13^C NMR (CDCl_3_, 75 MHz): *δ* 203.3, 203.2, 173.3, 146.7, 146.6, 138.2, 135.6, 128.8, 128.5, 128.4, 127.7, 127.3, 127.2, 124.3, 124.2, 121.3, 118.9, 118.7, 111.2, 110.7, 65.8, 62.6, 61.7, 61.5, 56.1, 54.8, 51.4, 46.4, 46.1, 25.7, 6.0, 5.8; FTIR (KBr, pellet): 3414, 1732, 1620, 1602, 1494, 1454 cm^−1^; ESI-MS *m*/*z*: 911 [M+H]^+^, 933 [M+Na]^+^. HRMS calculated for C_54_H_59_N_10_O_4_ (MH^+^) 911.4715; found: 911.4705.

Ethyl* N*-((1-((*R*)-3-(dibenzylamino)-2-oxobutyl)-1*H*-1,2,3-triazol-4-yl)methyl)-N-((1-(3-(dibenzylamino)-2-oxobutyl)-1*H*-1,2,3-triazol-4-yl)methyl)glycinate (**6c**). Yield 50%; [*α*]^20^_*D*_ = −5.6 (*c* = 0.14 in CH_2_Cl_2_); ^1^H NMR (CDCl_3_, 200 MHz): *δ* 7.81–7.23 (m, 22H), 5.72 (d, 2H, *J* = 18.8 Hz), 5.15 (d, 2H, *J* = 18.8 Hz), 3.81–3.34 (m, 18H), 1.23 (s, 9H); ^13^C NMR (CDCl_3_, 75 MHz): *δ* 203.7, 171.3, 144.0, 138.3, 128.9, 128.8, 127.7, 125.3, 63.2, 61.5, 60.8, 56.5, 54.9, 47.9, 14.2, 6.1; ESI-MS *m*/*z*: 796 [M+H]^+^, 818 [M+Na]^+^.

Ethyl* N*-((1-((*R*)-3-(dibenzylamino)-2-oxobutyl)-1*H*-1,2,3-triazol-4-yl)methyl)-*N*-((1-((*S*)-3-(dibenzylamino)-2-oxobutyl)-1*H*-1,2,3-triazol-4-yl)methyl)-*D*-serinate (**6d**). Yield 55%; pale yellow solid; ^1^H NMR (CDCl_3_, 200 MHz): *δ* 7.38–7.27 (m, 22H), 5.65 (d, 2H, *J* = 17.6 Hz), 5.08 (d, 2H, *J* = 18.3 Hz), 4.18–3.51 (m, 20H), 1.27 (s, 9H); ^13^C NMR (CDCl_3_, 75 MHz): *δ* 203.1, 171.3, 146.1, 138.3, 128.9, 128.8, 127.7, 124.3, 64.9, 61.5, 60.8, 59.8, 56.4, 54.9, 46.4, 14.3, 6.0; ESI-MS *m*/*z*: 827 [M+H]^+^.

### 4.3. DPPH-Scavenging Activity

The DPPH-scavenging activities of the bis-(1,2,3-triazoles)amino esters** 6** were assessed as described by Sivakumar et al. [51] with slight modifications. This method is based on the reduction of DPPH in the presence of antioxidants; the antioxidant activity is detected as a change from purple to yellow color in the solution. Briefly, a solution of DPPH (0.10 mM) in methanol (grade HPLC) was prepared. Then, 50 *μ*L of the bis-(1,2,3-triazoles)amino esters (0.1–25 *μ*g mL^−1^ in methanol) was mixed with 1.950 mL of the DPPH solution and the mixture is incubated for 20 min at room temperature in darkness conditions, and the absorbance was measured at 517 nm (Spectronic Genesys 5). The DPPH-scavenging activity of the bis-(1,2,3-triazoles)amino esters** 6** was calculated as follows:

(1) $$\begin{array}{c} \text{DPPH-scavenging effect}\left(\%\right)=\left[\frac{\left(A_{0}-A_{1}\right)}{A_{0}}\right]\times 100, \end{array}$$

where *A*_0_ was the absorbance of control and *A*_1_ was the absorbance in the presence of the bis-(1,2,3-triazoles)amino esters. The same methodology was used for assaying the references standard vitamin C and gallic acid (0.1–25 *μ*g mL^−1^ in methanol). The bis-(1,2,3-triazoles)amino esters and reference standard are expressed as inhibitory concentration mean (IC_50_). Linear regression analysis was used to calculate the IC_50_ values and corresponded to the mean ± standard deviation of one experiment by triplicate and was determined by SPSS Statistics software v19 (IBM company).

## Supplementary Material

This supplementary material contains experimental procedures for synthesis of *α*-halomethyl ketones and *N*,*N*-dipropargylamino acids. The spectroscopic data for all compounds is included.

## Figures and Tables

**Table 1:** *α*-Halomethyl ketones synthesized from *α*-amino acids.

Compound	R^1^	X	Yield (%)
**10a**	–CH_3_	Cl	86
**10b**	–H	Cl	80
**10c**	–CH_2_Ph	Cl	90
**10d**	TBDMSOCH_2_–	Cl	65
**10e**	–CH_3_	Br	90
**10f**	–H	Br	83

**Table 2:** Dialkynes synthesized from *α*-amino acids.

Compound	R^1^	R^2^	Yield (%)
**11a**	–CH_3_	–CH_3_	85
**11b**	–H	–CH_2_–CH_3_	67
**11c**	–CH_2_Indol	–CH_3_	87
**11d**	–CH_2_Ph	–CH_3_	74
**11e**	–CH_2_OH	–CH_2_–CH_3_	59

**Table 3:** Yield and antioxidant activity of bis-(1,2,3-triazolylmethyl)amino esters **6**.

Product	R^1^	R^2^	Yield %	Antioxidant activity IC_50_ (*µ*g mL^−1^)^a^
**6a**	–CH_3_	–CH_3_	65	317.68 ± 74.16
**6b**	–CH_2_Indol	–CH_3_	76	774.26 ± 112.64
**6c**	–H	–CH_2_CH_3_	50	75.57 ± 1.74
**6d**	–CH_2_OH	–CH_2_CH_3_	55	—
Vitamin C			—	8.48 ± 1.07
Gallic acid			—	6.33 ± 0.66

^a^Values represent mean ± standard deviation, *n* = 3.
