# Supplementary material for: Synthesis and Antioxidant Evaluation of Enantiomerically Pure Bis-(1,2,3-triazolylmethyl)amino Esters from Modified α-Amino Acids
Source: ScientificWorldJournal. 2014 Oct 15;2014:264762. doi: 10.1155/2014/264762 (PMC4216715; doi:10.1155/2014/264762)
Supplement: Supplementary file 1 — This supplementary material contains experimental procedures for synthesis of α-halomethyl ketones and N,N-dipropargylamino acids. The spectroscopic data for all compounds is included. [file 264762.f1.pdf]

Supplementary Information

Synthesis and antioxidant evaluation of enantiomerically pure bis-(1,2,3-triazolylmethyl)amino esters from modified α-amino acids

Juan I. Sarmiento-Sánchez[a]*, Adrián Ochoa-Teran[b] and Ignacio A. Rivero[b]*

[a]Facultad de Ingeniería, Universidad Autónoma de Sinaloa, Blvd. de las Américas, S/N, Culiacán, Sinaloa, C.P. 80000, Mexico

[b]Centro de Graduados e Investigación del Instituto Tecnológico de Tijuana, Apdo. Postal 1166, 22500, Tijuana, B.C., Mexico

* authors with equal contribution. Corresponding address: Facultad de Ingeniería, Universidad Autónoma de Sinaloa, Blvd. de las Américas, S/N, 80040, Culiacán, Sinaloa, México. Tel. +52 (667) 7134043. E-mail address: jsarmiento@uas.edu.mx or irivero@tectijuana.mx

CONTENIDO

GENERAL PROCEDURE FOR THE SYNTHESIS OF A-HALOMETHYL KETONES.................... 3

GENERAL PROCEDURE FOR THE SYNTHESIS OF DI-PROPARGYL A-AMINO ESTERS 4

FIGURE S1 - (*S*)-METHYL 2-(DI(PROP-2-YN-1-YL)AMINO)PROPANOATE (11A)........ 6

FIGURE S2 - ETHYL 2-(DI(PROP-2-YN-1-YL)AMINO)ACETATE (11B)........ 7

FIGURE S3 - (*S*)-METHYL 2-(DI(PROP-2-YN-1-YL)AMINO)-3-(1*H*-INDOL-3-YL)PROPANOATE (11C)........ 8

FIGURE S4 - (*S*)-METHYL 2-(DI(PROP-2-YN-1-YL)AMINO)-3-PHENYLPROPANOATE (11D)........ 9

FIGURE S5 - (*S*)-METHYL 2-(DI(PROP-2-YN-1-YL)AMINO)-3-HYDROXYPROPANOATE (11E)........ 10

FIGURE S6 - (S)-METHYL 2-(((1-((R)-3-(DIBENZYLAMINO)-2-OXOBUTYL)-1H-1,2,3-TRIAZOL-4-YL)METHYL)((1-((S)-3-(DIBENZYLAMINO)-2-OXOBUTYL)-1H-1,2,3-TRIAZOL-4-YL)METHYL)AMINO)PROPANOATE (6A)........ 11

FIGURE S7 - (R)-METHYL 2-(((1-((R)-3-(DIBENZYLAMINO)-2-OXOBUTYL)-1H-1,2,3-TRIAZOL-4-YL)METHYL)((1-((S)-3-(DIBENZYLAMINO)-2-OXOBUTYL)-1H-1,2,3-TRIAZOL-4-YL)METHYL)AMINO)-3-(1H-INDOL-3-YL)PROPANOATE (6B)........ 13

FIGURE S8 - ETHYL 2-(((1-((R)-3-(DIBENZYLAMINO)-2-OXOBUTYL)-1H-1,2,3-TRIAZOL-4-YL)METHYL)((1-((S)-3-(DIBENZYLAMINO)-2-OXOBUTYL)-1H-1,2,3-TRIAZOL-4-YL)METHYL)AMINO)ACETATE (6C)........ 15

FIGURE S9 - (R)-METHYL 2-(((1-((R)-3-(DIBENZYLAMINO)-2-OXOBUTYL)-1H-1,2,3-TRIAZOL-4-YL)METHYL)((1-((S)-3-(DIBENZYLAMINO)-2-OXOBUTYL)-1H-1,2,3-TRIAZOL-4-YL)METHYL)AMINO)-3-HYDROXY PROPANOATE (6D).. 16

1 General procedure for the synthesis of α-halomethyl ketones

In a round bottom flask equipped with an addition funnel was added α-dibenzylamino ester (1 equiv), chloroiodomethane or dibromomethane (2.5 equiv), 50 mL of anhydrous THF at -78 °C under inert atmosphere (argon) with constant stirring. Next, a solution of methyllithium 1.5 M (2.5 equiv) was added dropwise and then allowed to react for 30 min at -78 °C with constant stirring. A saturated solution of ammonium chloride (20 mL) was then added and kept with stirring until it reached room temperature. The product was extracted with diethyl ether (3 x 20 mL) and the extracts were combined and dried over anhydrous sodium sulfate. After evaporation of the solvent, the resulting oil was used for the next step.

(*S*)-1-chloro-3-(dibenzylamino)-2-butanone (**10a**). For full characterization see (Barluenga et al., 1995), yield 86%; pale yellow oil; ^{1}H NMR ($CDCl_3$, 200 MHz): δ 7.35-7.23 (m, 10H), 4.56 (d, 1H, *J* = 15.9 Hz), 4.20 (d, 1H, *J* = 15.9 Hz), 3.70 (d, 2H, *J* = 13.6 Hz), 3.58-3.48 (m, 1H), 3.42 (d, 2H, *J* = 13.6 Hz), 1.22 (d, 3H, *J* = 6.6 Hz); ^{13}C NMR ($CDCl_3$, 50 MHz): δ 203.0, 138.4, 128.7, 128.6, 127.5, 61.0, 54.6, 47.8, 6.5.

1-chloro-3-(dibenzylamino)-2-propanone (**10b**). Yield 80%; pale yellow oil; ^{1}H NMR ($CDCl_3$, 200 MHz): δ 7.35-7.26 (m, 10H), 4.14 (s, 2H), 3.66 (s, 4H), 3.34 (s, 2H); 13C NMR (CDCl3, 50 MHz): δ 201.8, 138.0, 129.8, 128.5, 127.5, 60.1, 59.1, 47.7.

(*S*)-1-chloro-3-(dibenzylamino)-4-phenyl-2-butanone (**10c**). For full characterization see ref.(Barluenga et al., 1995), yield 90%; pale yellow oil; ^{1}H NMR($CDCl_3$, 200 MHz): δ 7.46-7.11 (m, 15H) , 4.43 (d, 2H, *J* = 15.9 Hz), 3.82 (d, 2H, *J* = 12.7 Hz), 3.66 (dd, 1H, *J* = 3.7, 9.3 Hz), 3.55 (d, 2H, *J* = 13.5 Hz), 3.27-3.16 (dd, 1H, *J* = 9.4, 13.3 Hz), 3.01-2.93 (dd, 1H, *J* = 3.7, 13.2 Hz); ^{13}C-NMR (CDCl3, 50 MHz): δ 200.5, 138.6, 138.4, 129.4, 128.8, 128.6, 128.4, 127.5, 126.2, 66.6, 54.5, 47.8, 28.8.

(*S*)-4-(tert-butildimethylsilyloxi)-1-chloro-3-(dibenzylamino)-2-butanone (**10d**). For full characterization see (Barluenga et al., 1999), yield 65%; pale yellow oil; ^{1}H NMR ($CDCl_3$, 200 MHz): δ 7.34-7.21 (m, 10H), 4.74 (s, 2H), 4.35 (d, 2H, *J* = 14.3 Hz), 4.09 (d, 2H, *J* =13.9 Hz), 3.70-3.60 (dd, 1H, *J* = 6.6 Hz), 0.85 (s, 9H), -0.02 (s, 6H); ^{13}C-NMR ($CDCl_3$, 50 MHz): δ 201.7, 139.0, 128.9, 128.5, 127.3, 65.4, 64.9, 55.3, 48.3, 25.9, 18.1, -5.6, -5.3.

(*S*)-1-bromo-3-(dibenzylamino)-2-butanone (**10e**). For full characterization see ref. (Barluenga et al., 1999), yield 90%; pale yellow oil; ^{1}H NMR (DMSO, 200 MHz): δ 7.46-7.18 (m, 10H), 4.27 (d, 1H, *J* = 13.2 Hz), 4.19 (d, 1H, *J* = 13.2 Hz), 3.66-3.78 (m, 3H), 3.48 (d, 2H, *J* = 6.7 Hz), 1.27 (d, 3H, *J* = 6.7 Hz); ^{13}C NMR (DMSO, 50 MHz): δ 202.2, 138.8, 128.4, 128.0, 126.3, 60.9, 53.8, 35.1, 7.6

1-bromo-3-(dibenzylamino)-2-propanone (**10f**). Yield 80%; pale brown oil; ^{1}H NMR ($CDCl_3$, 200 MHz): δ 7.41-7.22 (m, 10H), 3.89 (s, 2H), 3.67 (s, 4H), 3.39 (s, 2H); ^{13}C NMR ($CDCl_3$, 50 MHz): δ 201.6, 138.0, 129.0, 128.5, 127.5, 60.7, 59.0, 32.8.

2 General procedure for the synthesis of di-propargyl α-amino esters

In a round bottom flask was added α-amino ester hydrochloride (1 equiv), potassium iodide (0.5 equiv), sodium bicarbonate (2.5 equiv) and propargyl chloride (2.5 equiv) in 50 mL of THF/DMSO (4:1 v/v) and refluxed for 8 h with constant stirring. The reaction mixture was transferred to a separating funnel, added water (250 mL) and extracted with DCM (3 x 60 mL). The extracts were combined and dried over anhydrous sodium sulfate. After evaporation of the solvent, the resulting oil was purified by flash chromatography.

(*S*)-methyl 2-(di(prop-2-yn-1-yl)amino)propanoate (**11a**). Yield 85%; yellow oil; R_f 0.40 (ethyl acetate/petroleum ether 2:8 v/v); ^{1}H NMR ($CDCl_3$, 200 MHz): δ 3.72 (s, 3H), 3.67-3.62 (m, 1H), 3.60 (d, 4H, *J* = 2.5 Hz), 2.26 (t, 2H, *J* = 2.3 Hz), 1.38 (d, 3H, *J* = 6.9 Hz). ^{13}C NMR ($CDCl_3$, 50 MHz): δ 173.5, 78.9, 73.1, 58.9, 51.7, 39.7, 15.8; IR (film): 3290, 2120, 2106, 1732, 1436 cm^{-1}; ESI-MS m/z: 218 $[M+K]^+$

Ethyl 2-[di(prop-2-yn-1-yl)amino]acetate (**11b**). Yield 67%, yellow oil, R_f 0.36 (ethyl acetate/petroleum ether 2:8 v/v); ^{1}H NMR ($CDCl_3$, 200 MHz): δ 4.25-4.15 (q, 2H, *J* = 6.9 Hz), 3.57 (d, 4H, *J* = 2.2 Hz), 3.45 (s, 2H), 2.29-2.27 (t, 2H, *J* = 2.3 Hz), 1.32-1.25 (t, 3H, *J* = 7.1 Hz); ^{13}C NMR ($CDCl_3$, 50 MHz): δ 170.2, 78.2, 73.7, 60.8, 53.5, 42.6, 14.2; FT-IR (film): 3290, 2120, 2106, 1744, 1444 cm^{-1}; ESI-MS m/z: 218 $[M+K]^+$

Methyl (*S*)-2-[di(prop-2-yn-1-yl)amino]-3-(1*H*-indol-3-yl)propanoate (**11c**). Yield 87%; brown solid; m.p. 55-57 °C; R_f 0.38 (ethyl acetate/petroleum ether 1:1 v/v); ^{1}H NMR ($CDCl_3$, 200 MHz): δ 8.12 (s, 1H), 7.67-7.58 (m, 1H), 7.36-6.98 (m, 4H), 3.91-3.81 (dd, 1H, *J* = 5.9 Hz), 3.72 (d, 4H, *J* = 2.2 Hz), 3.53 (s, 3H), 3.28-3.18 (m, 2H), 2.27 (t, 2H, *J* = 2.2 Hz); ^{13}C NMR ($CDCl_3$, 50 MHz): δ 172.4, 136.1, 127.4, 122.8, 121.9, 119.4, 118.6, 111.2, 111.1, 79.3, 73.0, 64.6, 51.3, 39.9, 25.9; FT-IR (film): 3412, 3290, 2120, 2106, 1729, 1620, 1595, 1340, 1213, 1170 cm^{-1}; ESI-MS m/z: 318 $[M+Na]^+$, 333 $[M+K]^+$

Methyl (*S*)-2-[di(prop-2-yn-1-yl)amino]-3-phenylpropanoate (**11d**). Yield 74%; pale yellow oil; R_f 0.80 (ethyl acetate/petroleum ether 1:1 v/v); ^{1}H NMR ($CDCl_3$, 200 MHz): δ 7.44-7.07 (m, 5 H), 3.78-3.70 (t, 1H, *J* = 7.7 Hz), 3.67 (d, 4H, *J* = 2.2 Hz), 3.57 (s, 3 H), 3.04 (d, 2H, *J* = 7.7 Hz), 2.26-2.24 (t, 2H, *J* = 2.3 Hz); ^{13}C NMR ($CDCl_3$, 50 MHz): δ 171.9, 137.3, 129.2, 129.4, 126.6, 79.1, 73.0, 65.7, 51.3, 39.9, 36.29; FT-IR (film): 3290, 2120, 2108, 1730, 1604, 1168 cm^{-1}; ESI-MS m/z: 256 $[M+H]^+$, 278 $[M+Na]^+$

Methyl (*S*)-2-[di(prop-2-yn-1-yl)amino]-3-hydroxypropanoate **(11e).** Yield 59%; pale yellow oil; R_f 0.85 (ethyl acetate/petroleum ether 1:1 v/v); ^{1}H NMR ($CDCl_3$, 200 MHz): δ 4.24-4.13 (q, 2H, *J* = 7.1 Hz), 3.88-3.74 (m, 2H), 3.68-3.66 (t, 4H, *J* = 2.2 Hz), 2.68-2.64 (m, 1H), 2.30-2.28 (t, 2H, *J* = 2.3 Hz), 1.34-1.27 (t, 3H, *J* = 7.1 Hz); ^{13}C NMR ($CDCl_3$, 50 MHz): δ 170.6, 79.3, 73.2, 64.1, 60.9, 59.8, 40.1, 14.2. IR (film): 3416, 3290, 2120, 2108, 1730, 1188 cm^{-1}; ESI-MS m/z: 210 $[M+H]^+$, 232 $[M+Na]^+$, 441 $[2M+Na]^+$

Figure S1 - (*S*)-methyl 2-(di(prop-2-yn-1-yl)amino)propanoate (11a).

^{1}H-NMR

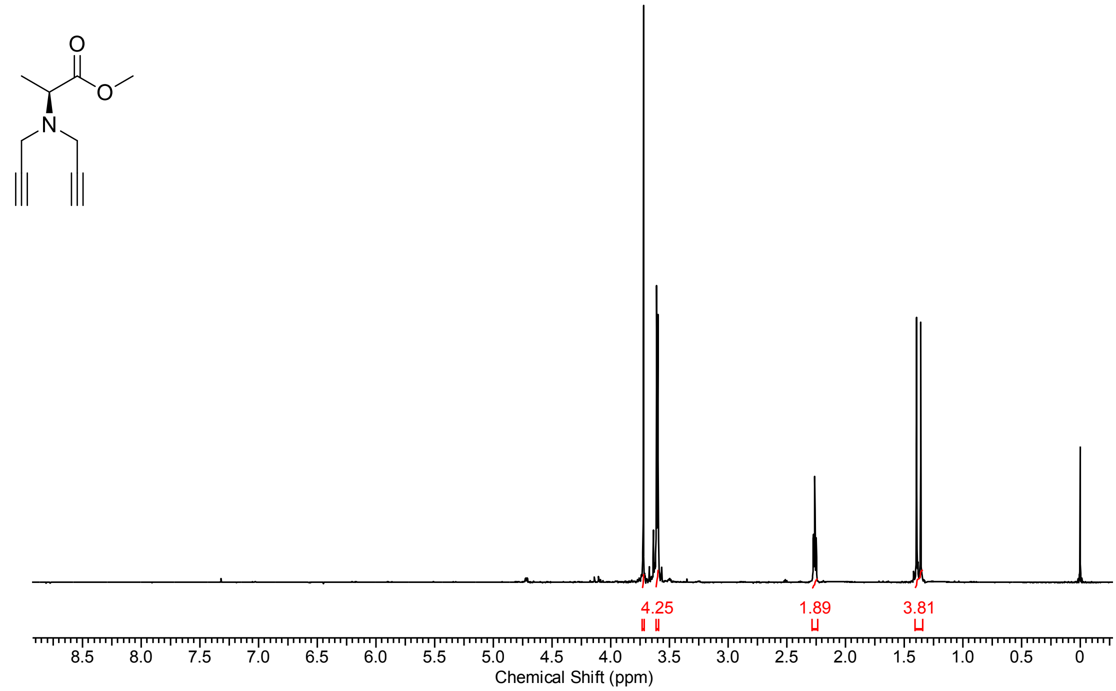

^{13}C-NMR

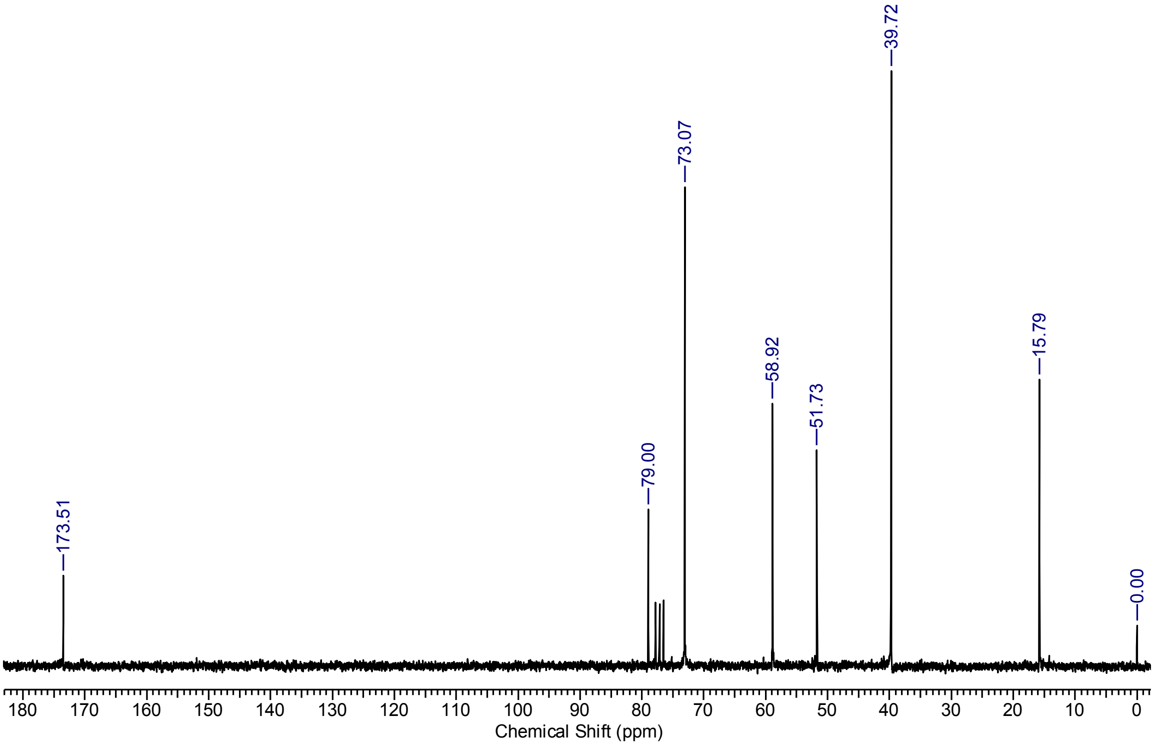

Figure S2 - Ethyl 2-(di(prop-2-yn-1-yl)amino)acetate (11b).

^{1}H-NMR

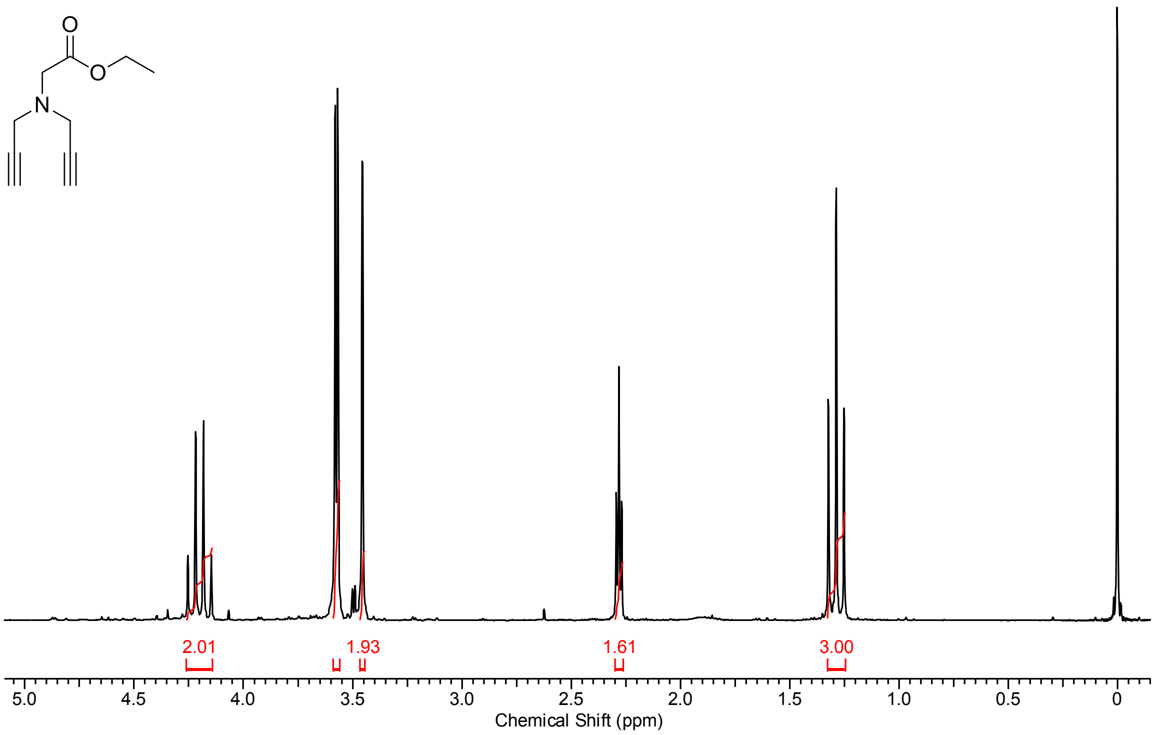

^{13}C-NMR

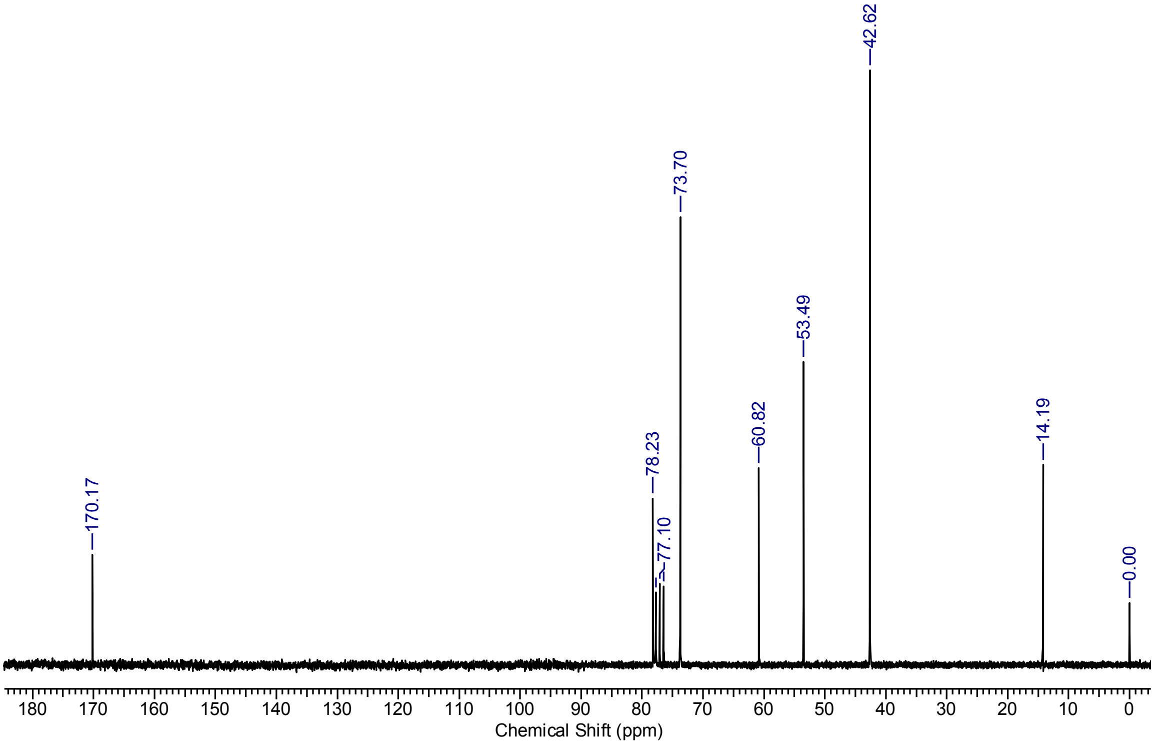

Figure S3 - (*S*)-methyl 2-(di(prop-2-yn-1-yl)amino)-3-(1*H*-indol-3-yl)propanoate (11c).

^{1}H-NMR

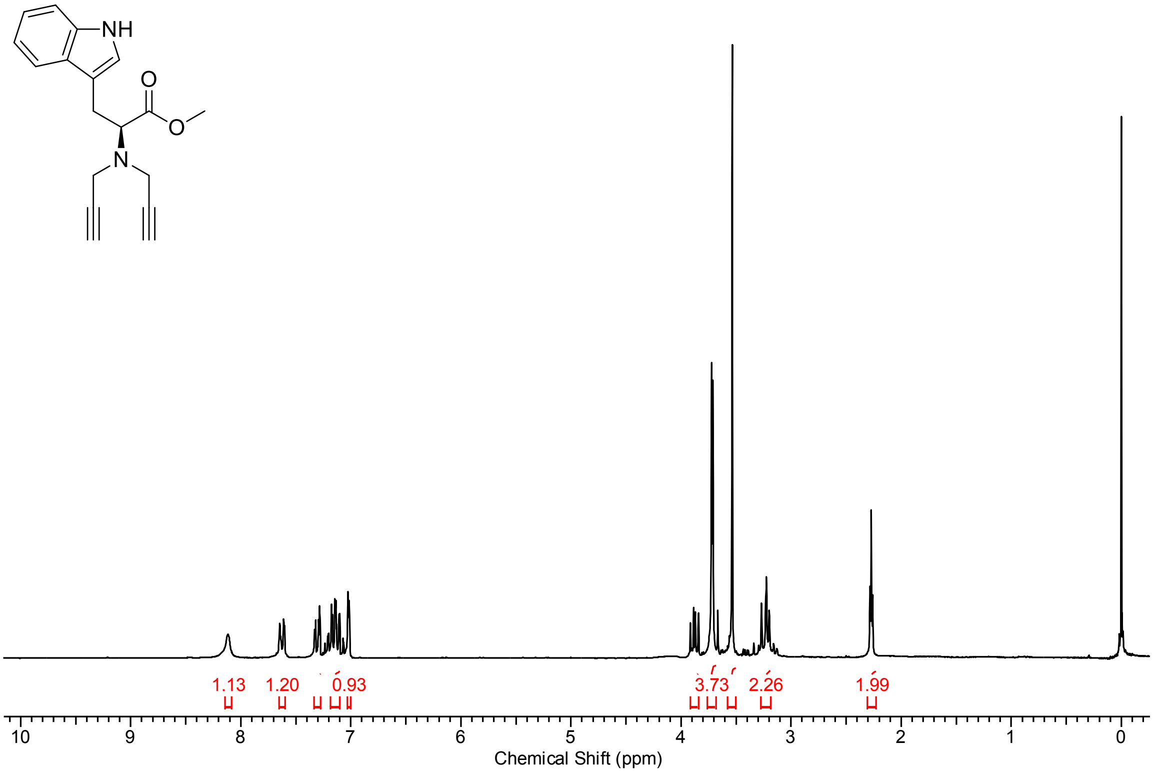

^{13}C-NMR

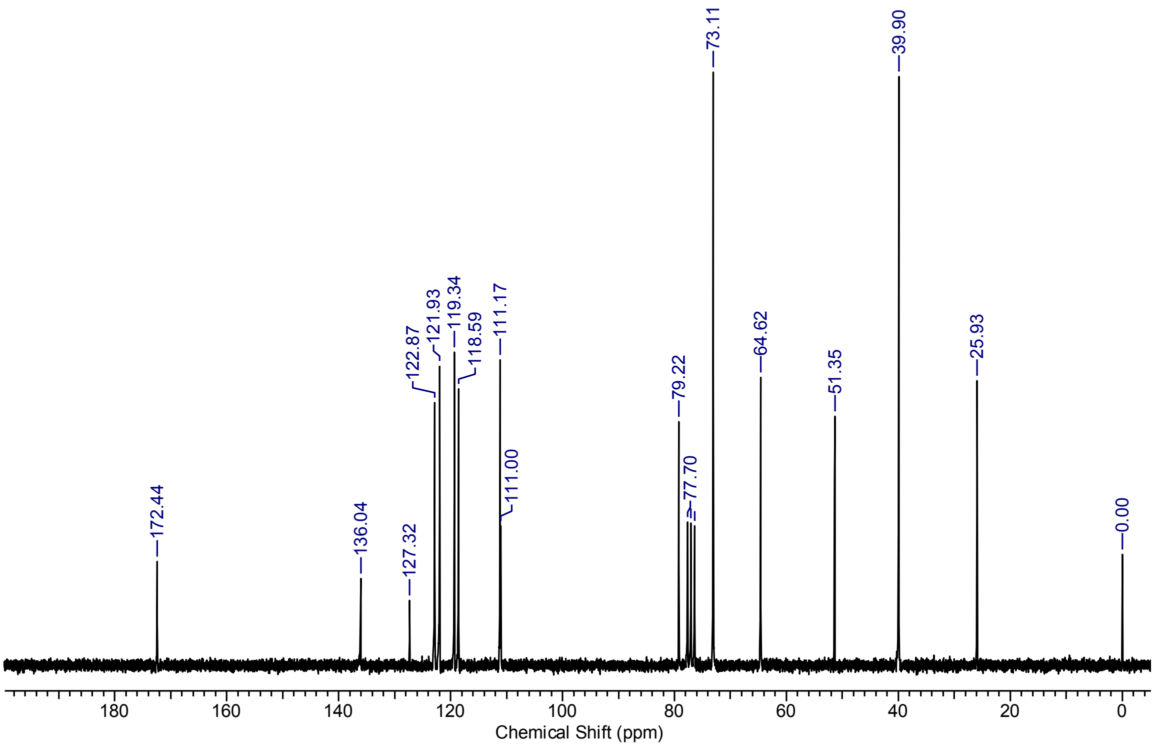

Figure S4 - (*S*)-methyl 2-(di(prop-2-yn-1-yl)amino)-3-phenylpropanoate (11d).

^{1}H-NMR

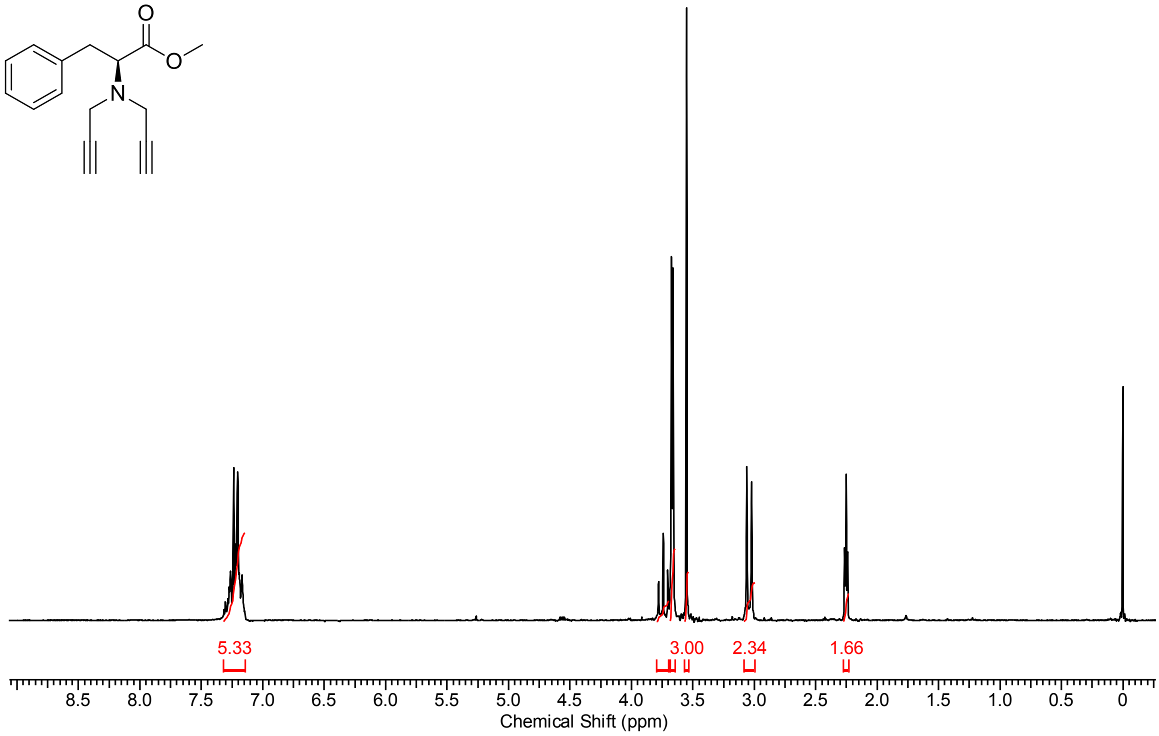

^{13}C-NMR

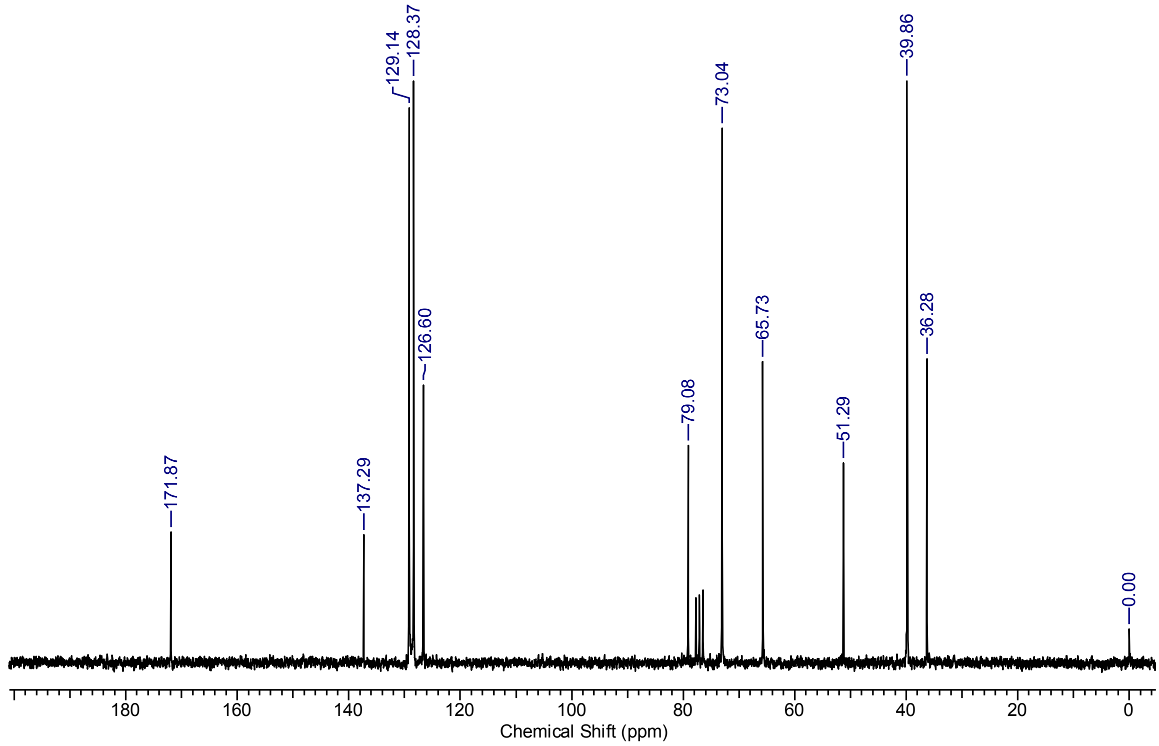

Figure S5 - (*S*)-methyl 2-(di(prop-2-yn-1-yl)amino)-3-hydroxypropanoate (11e).

^{1}H-NMR

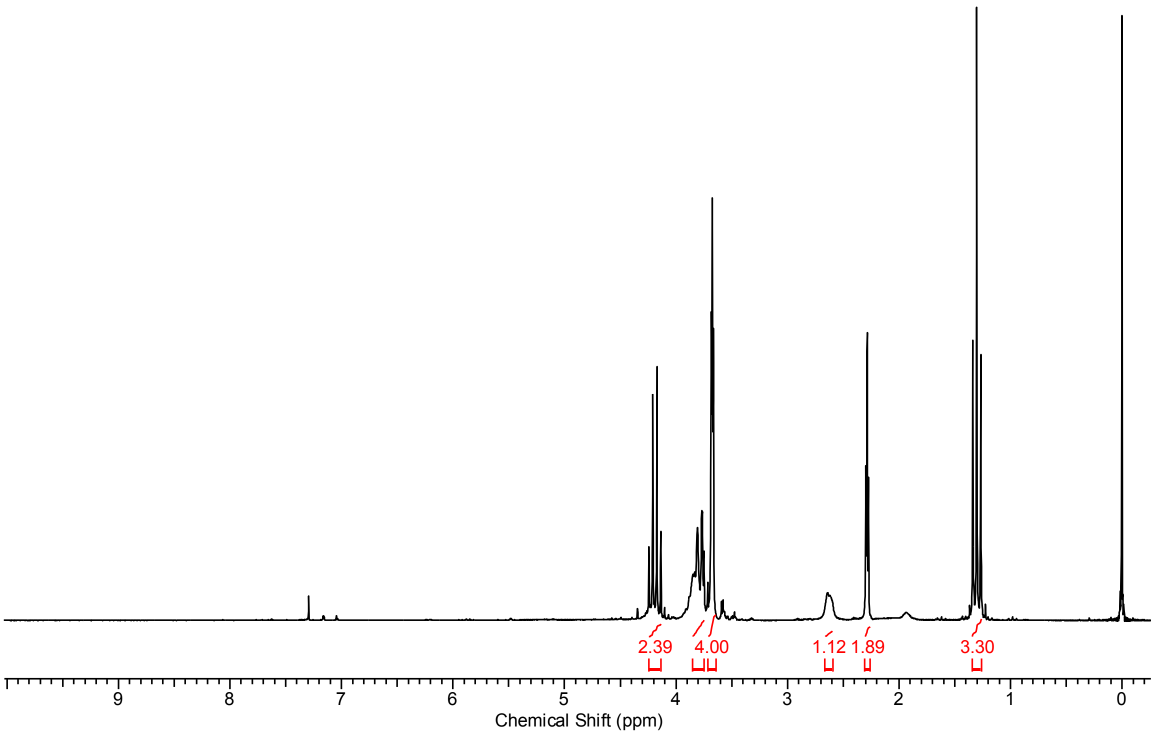

^{13}C-NMR

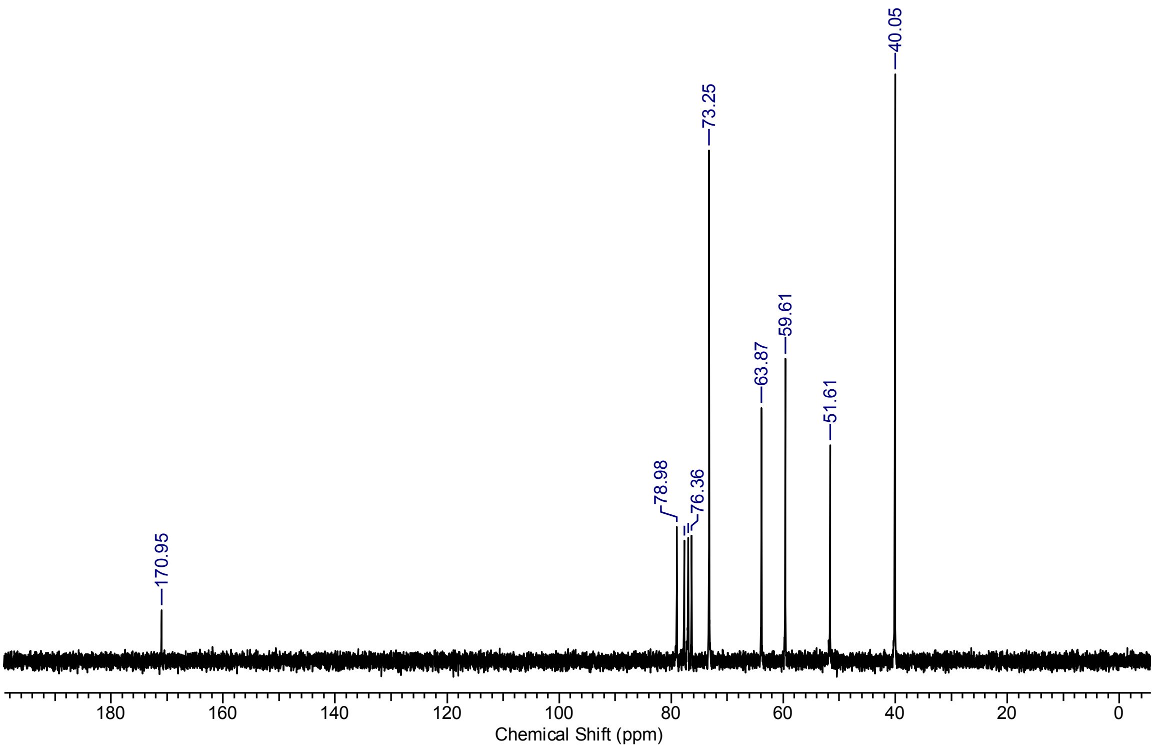

Figure S6 - (S)-methyl 2-(((1-((R)-3-(dibenzylamino)-2-oxobutyl)-1H-1,2,3-triazol-4-yl)methyl)((1-((S)-3-(dibenzylamino)-2-oxobutyl)-1H-1,2,3-triazol-4-yl)methyl)amino)propanoate (6a).

^{1}H-NMR

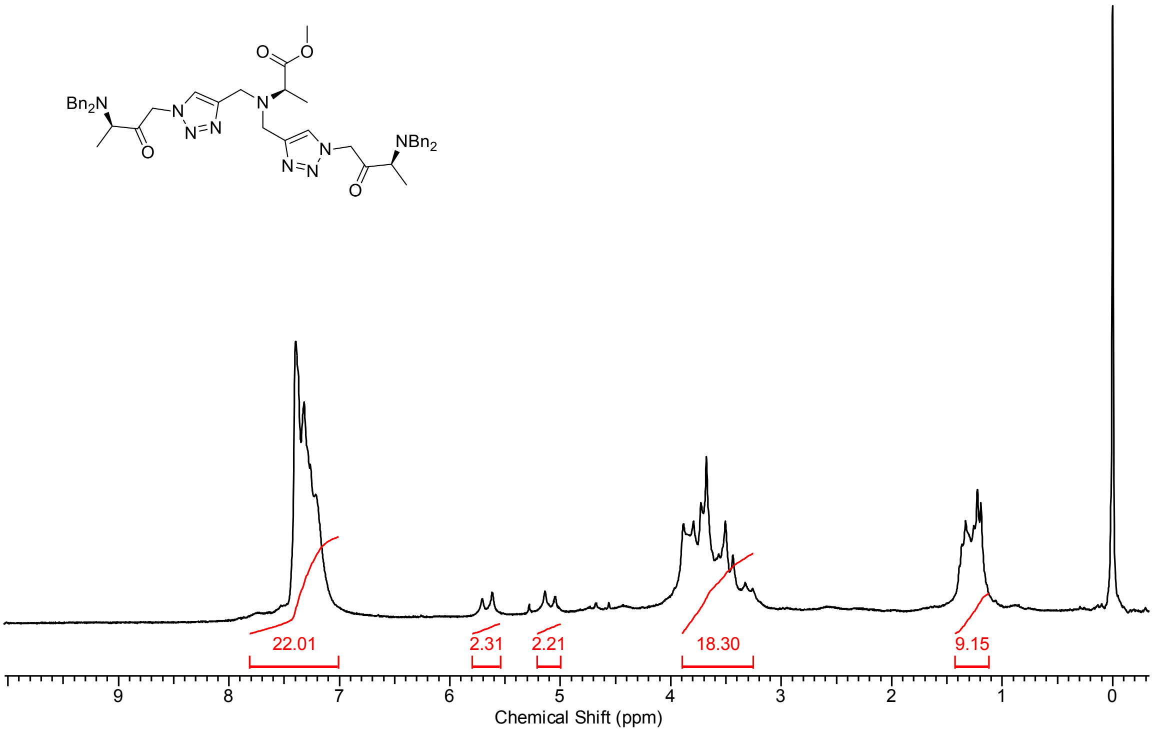

^{13}C-NMR

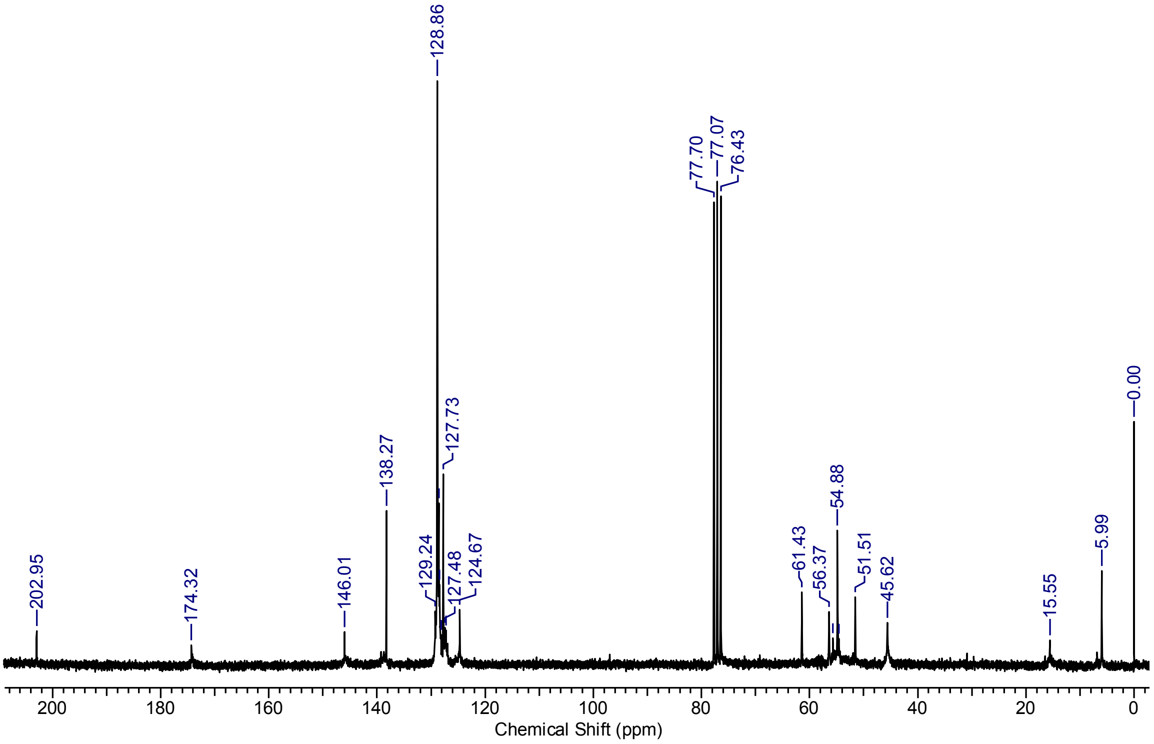

HRMS-ESI

Mass Spectrum SmartFormula Report

Analysis Info Acquisition Date 5/10/2014 2:08:57 PM

Analysis Name	D:\Data\Entrenamiento\Data\Juansarmiento\EXP171_MSPositivo_2.d		
Method	tune_low_LC_modificado.m	Operator	CBS_UAM_I
Sample Name		Instrument	micrOTOF 213750.00410
Comment			

Acquisition Parameter

Source Type	ESI	Ion Polarity	Positive	Set Nebulizer	5.0 Bar
Focus	Active			Set Dry Heater	190 °C
Scan Begin	50 m/z	Set Capillary	4500 V	Set Dry Gas	2.0 l/min
Scan End	2000 m/z	Set End Plate Offset	-500 V	Set Divert Valve	Source

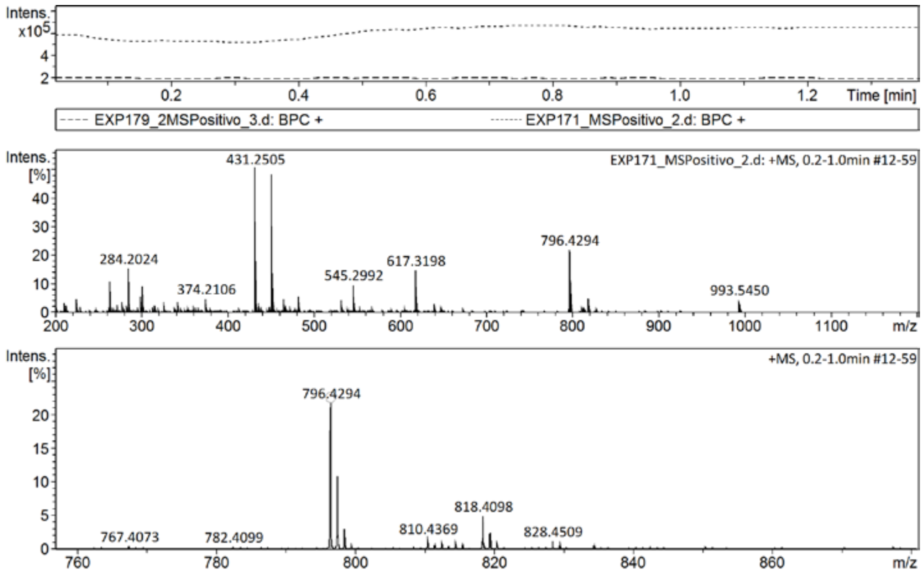

Meas. m/z	#	Ion Formula	m/z	err [ppm]	mSigma	# Sigma	Score	rdb	e^- Conf	N-Rule
796.4294	1	C46H54N9O4	796.4293	0.0	17.8	4	100.00	24.5	even	ok

Figure S7 - (R)-methyl 2-(((1-((R)-3-(dibenzylamino)-2-oxobutyl)-1H-1,2,3-triazol-4-yl)methyl)((1-((S)-3-(dibenzylamino)-2-oxobutyl)-1H-1,2,3-triazol-4-yl)methyl)amino)-3-(1H-indol-3-yl)propanoate (6b).

^{1}H-NMR

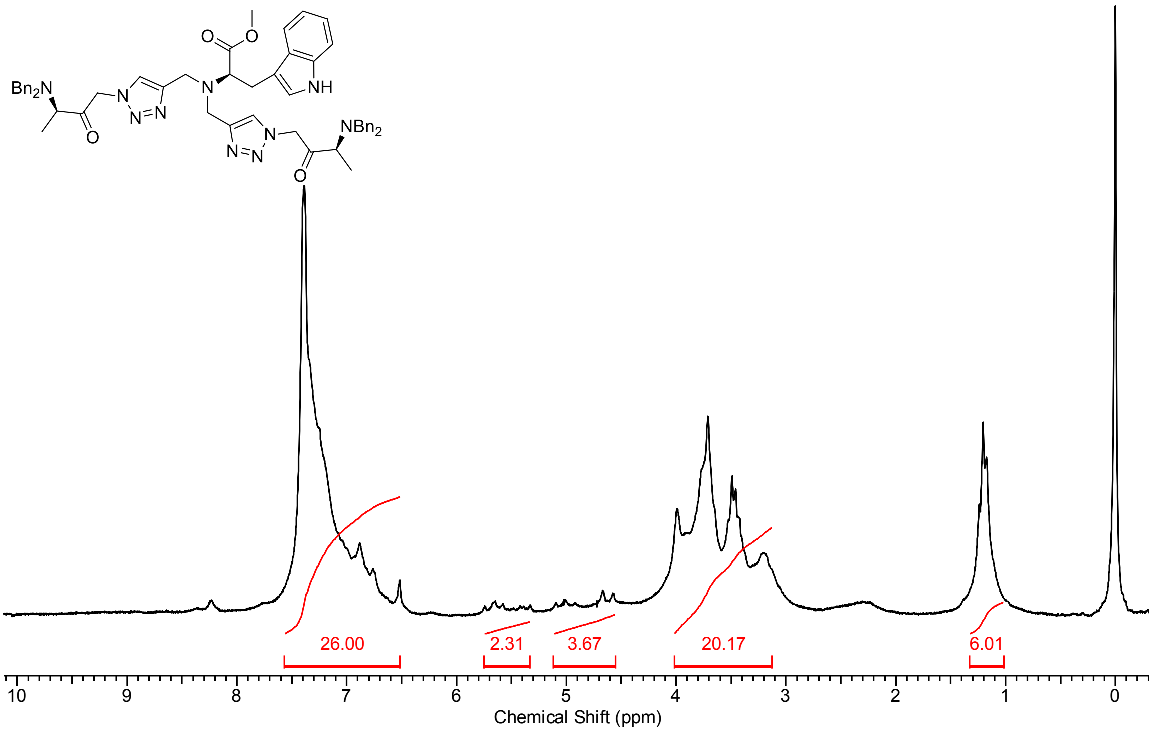

^{13}C-NMR

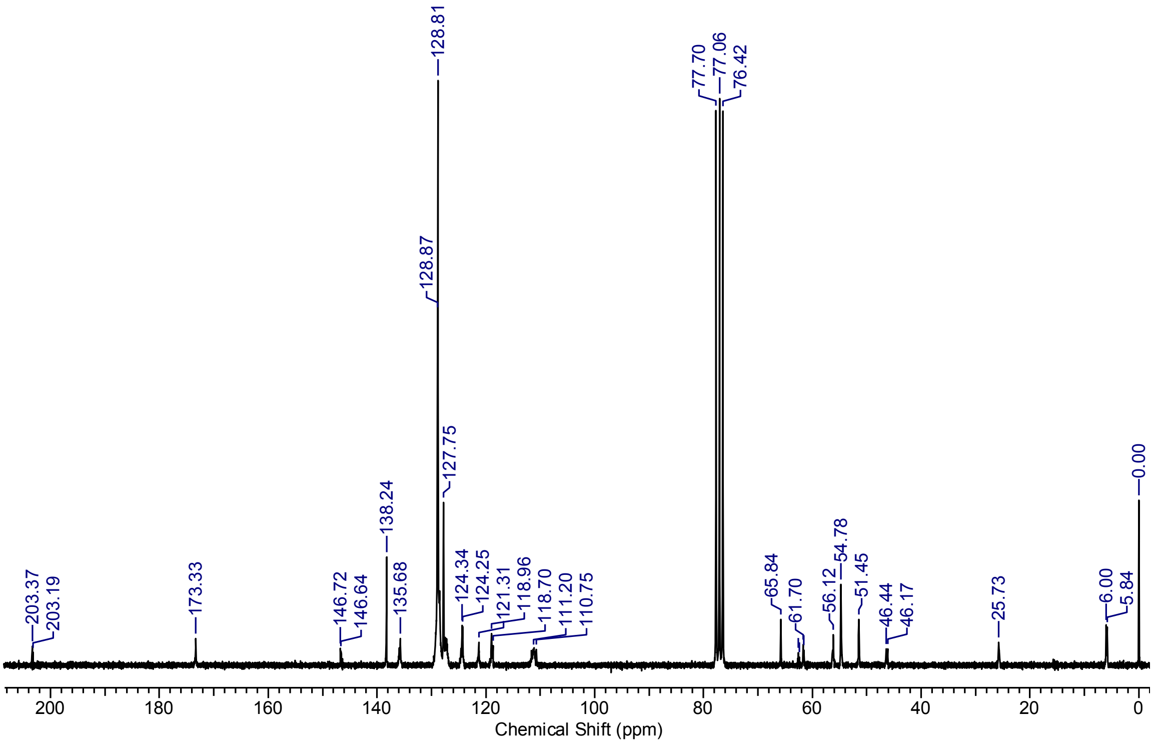

HRMS-ESI

Mass Spectrum SmartFormula Report

Analysis Info Acquisition Date 5/10/2014 1:53:42 PM

Analysis Name	D:\Data\Entrenamiento\Data\Juansarmiento\EXP179_2MSPositivo_3.d		
Method	tune_low_LC_modificado.m	Operator	CBS_UAM_I
Sample Name		Instrument	micrOTOF 213750.00410
Comment			

Acquisition Parameter

Source Type	ESI	Ion Polarity	Positive	Set Nebulizer	0.3 Bar
Focus	Active			Set Dry Heater	190 °C
Scan Begin	50 m/z	Set Capillary	4500 V	Set Dry Gas	3.0 l/min
Scan End	2000 m/z	Set End Plate Offset	-500 V	Set Divert Valve	Source

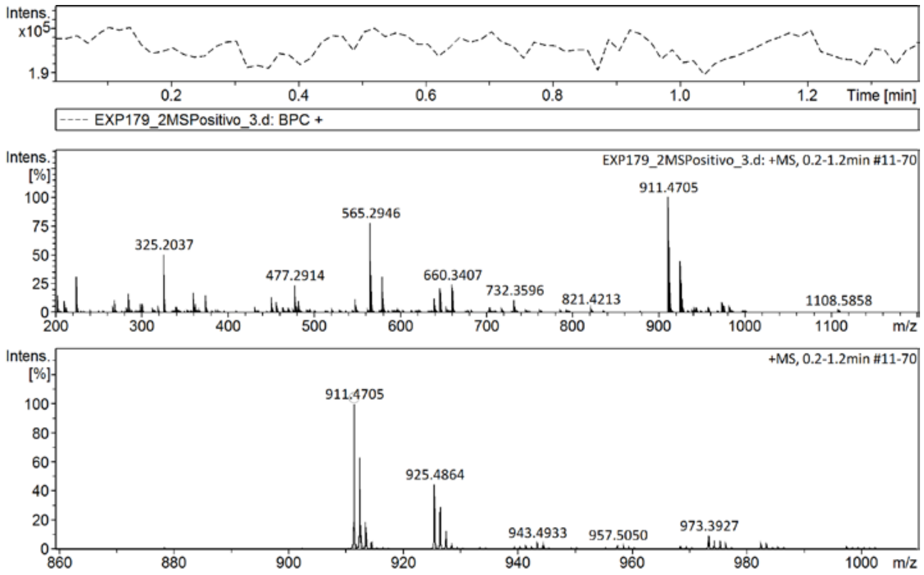

Meas. m/z	#	Ion Formula	m/z	err [ppm]	mSigma	# Sigma	Score	rdb	e⁻ Conf	N-Rule
911.4705	1	C54H59N10O4	911.4715	1.1	7.5	1	71.67	30.5	even	ok

Figure S8 - ethyl 2-(((1-((R)-3-(dibenzylamino)-2-oxobutyl)-1H-1,2,3-triazol-4-yl)methyl)((1-((S)-3-(dibenzylamino)-2-oxobutyl)-1H-1,2,3-triazol-4-yl)methyl)amino)acetate (6c).

^{1}H-NMR

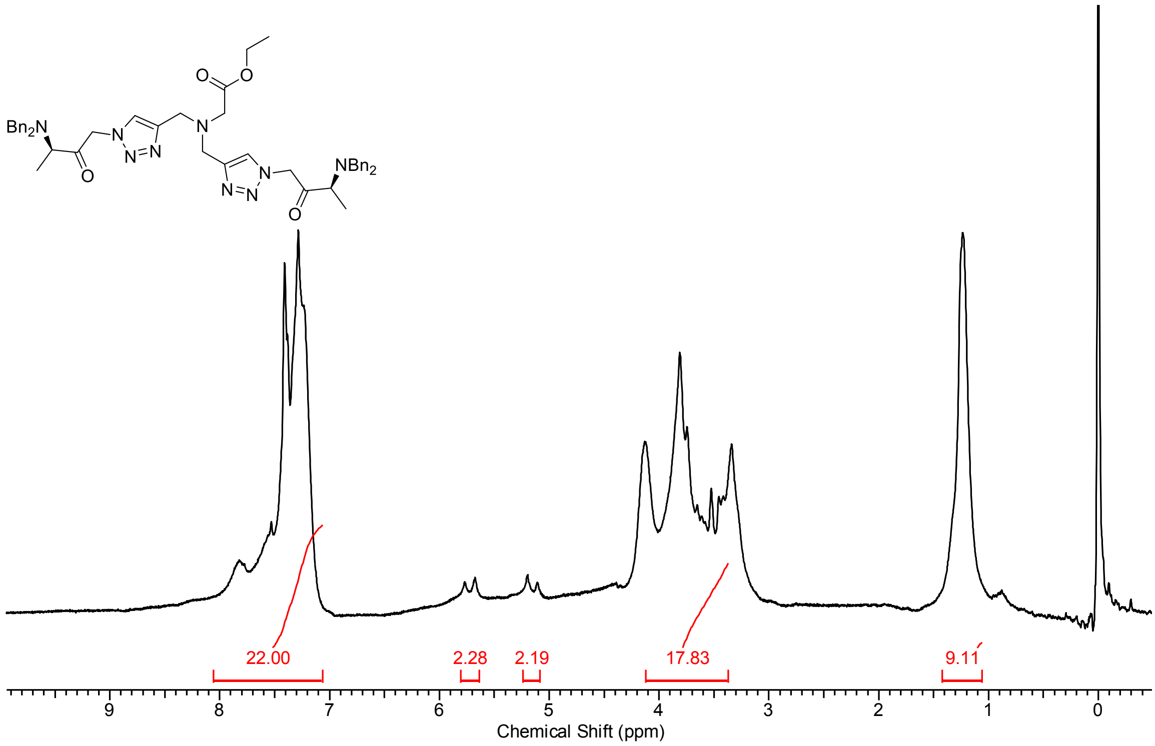

^{13}C-NMR

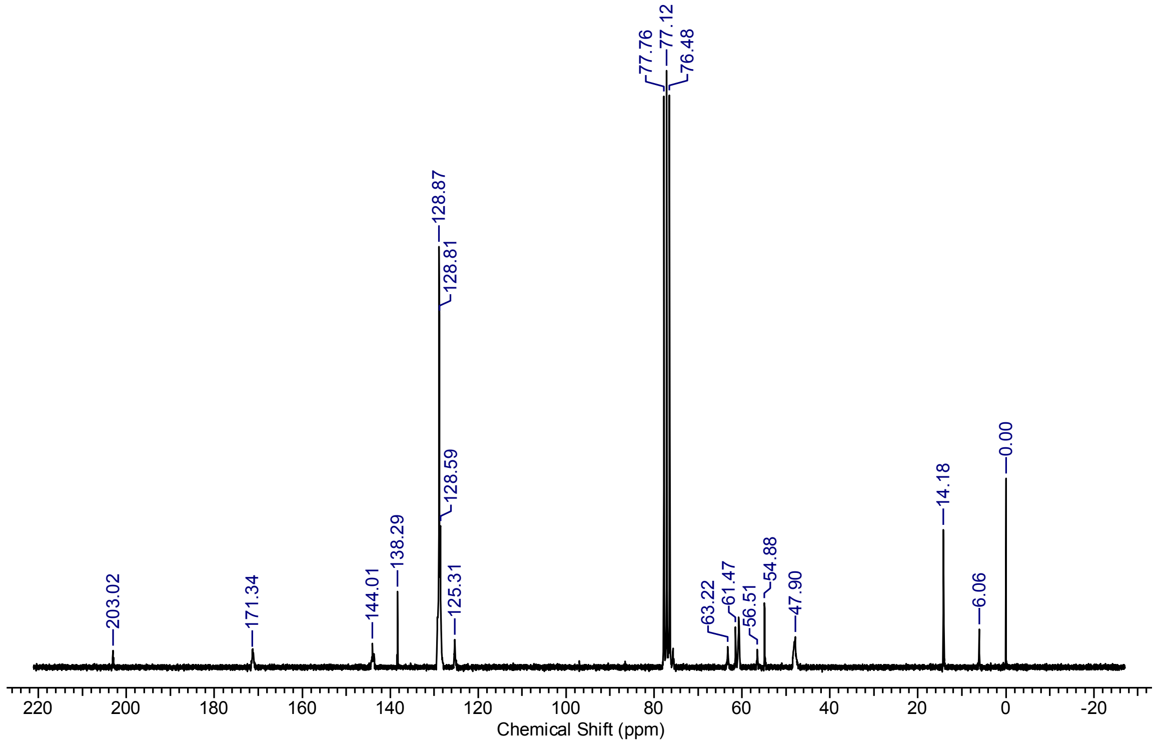

Figure S9 - (R)-methyl 2-(((1-((R)-3-(dibenzylamino)-2-oxobutyl)-1H-1,2,3-triazol-4-yl)methyl)((1-((S)-3-(dibenzylamino)-2-oxobutyl)-1H-1,2,3-triazol-4-yl)methyl)amino)-3-hydroxy propanoate (6d).

^{1}H-NMR

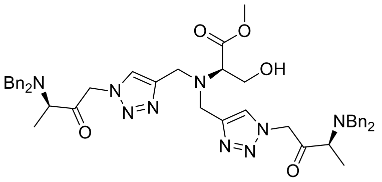

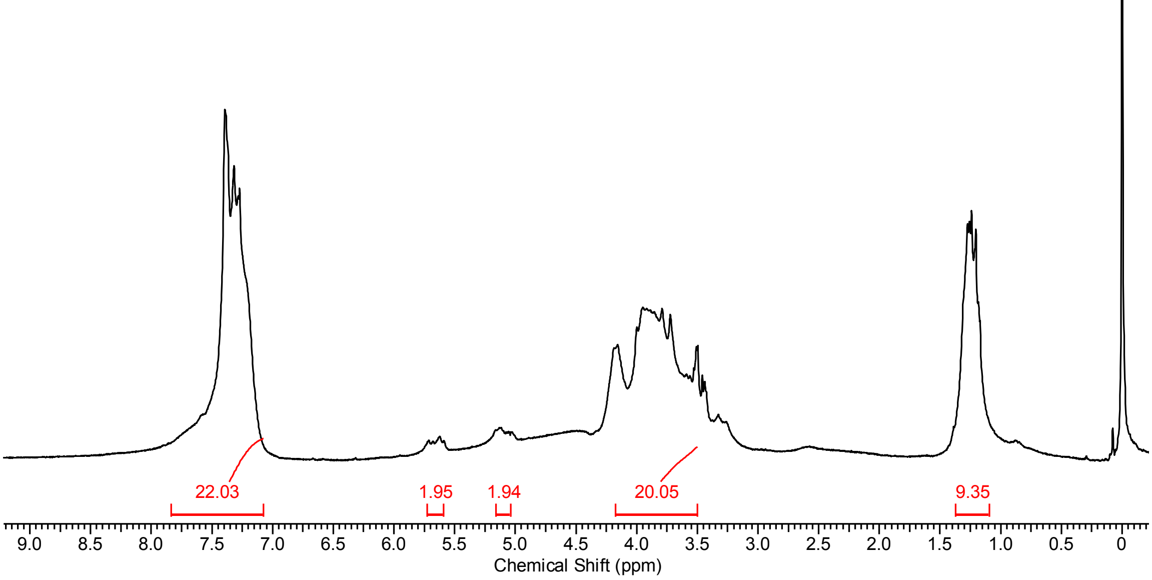

^{13}C-NMR

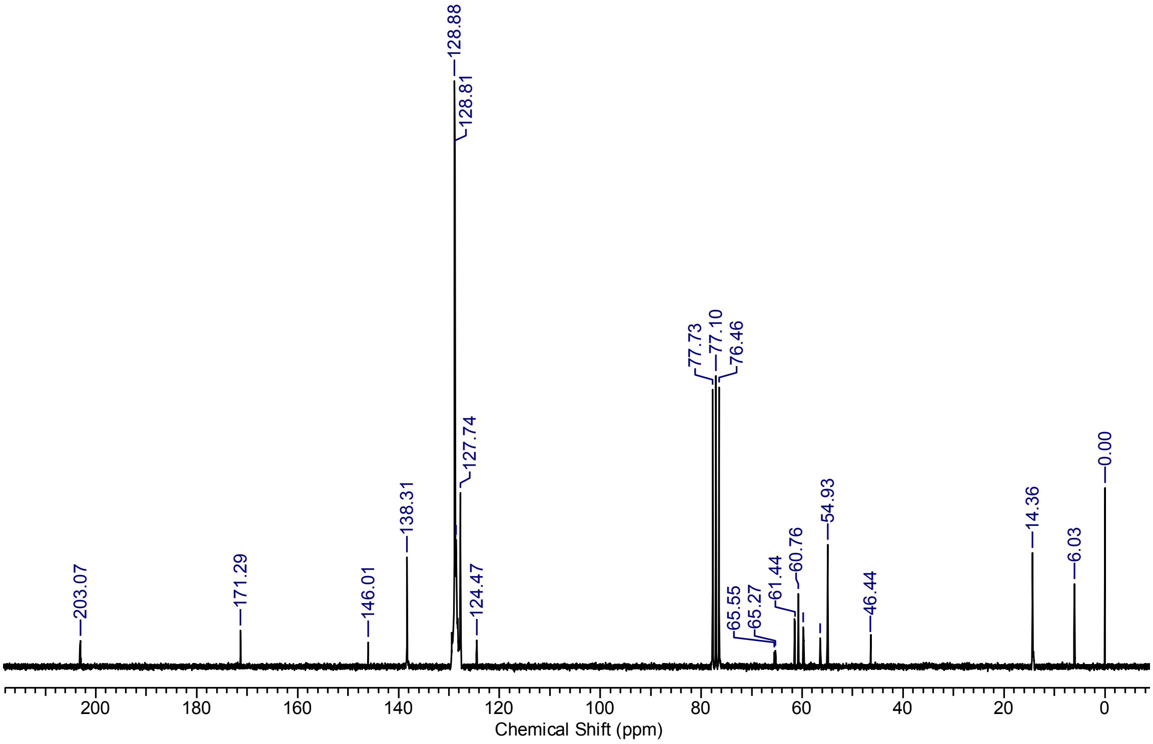

REFERENCES

Barluenga, J., Baragaña, B., Concellón, J.M., 1995. The Journal of Organic Chemistry 60 (21), 6696-6699.
Barluenga, J., Baragaña, B., Concellón, J.M., 1999. The Journal of Organic Chemistry 64 (8), 2843-2846.
